# MEWDS-like Presentation Unmasking Sequential Bilateral Multifocal Choroiditis: Insights from Longitudinal Multimodal Imaging

**DOI:** 10.3390/biomedicines14030649

**Published:** 2026-03-13

**Authors:** Blerta Lang, Annekatrin Rickmann, Karl Thomas Boden, Stefanie Behnke, Peter Szurman

**Affiliations:** 1 Eye Clinic Sulzbach, Knappschaft Hospitals Saar, An der Klinik 10, D-66280 Sulzbach, Germany; annekatrin.rickmann@knappschaft-kliniken.de (A.R.); karl.boden@knappschaft-kliniken.de (K.T.B.); peter.szurman@knappschaft-kliniken.de (P.S.); 2 Neurological Clinic Sulzbach, Knappschaft Hospitals Saar, An der Klinik 10, D-66280 Sulzbach, Germany; stefanie.behnke@knappschaft-kliniken.de; 3 Klaus Heimann Eye Research Institute, An der Klinik 10, D-66280 Sulzbach, Germany

**Keywords:** multifocal evanescent white dot syndrome, multifocal choroiditis, multimodal imaging, choriocapillaris, presumed retrobulbar neuritis, cystoid macular edema, adalimumab

## Abstract

**Background:** Multiple evanescent white dot syndrome (MEWDS) is usually acute and self-limited, whereas multifocal choroiditis (MFC)/punctate inner choroidopathy (PIC) is relapsing; overlap can obscure early diagnosis and requires longitudinal multimodal imaging. **Methods:** We report a 4-year follow-up of a 31-year-old woman with fundus autofluorescence (FAF), fluorescein angiography (FA), indocyanine green angiography (ICGA), and spectral-domain optical coherence tomography (SD-OCT), plus a systemic/neurologic/rheumatologic work-up. Treatment included intravenous methylprednisolone for presumed optic neuritis, followed by topical, periocular, intravitreal, and systemic corticosteroids, later escalated to adalimumab and an intravitreal dexamethasone implant. Because foveal granularity could not be documented, baseline was termed “MEWDS-like”. Diagnostic labelling was benchmarked against Standardization of Uveitis Nomenclature (SUN) criteria, and choroidal neovascularization (CNV) was assessed at each relapse by OCT and FA. **Results:** The right eye initially showed a MEWDS-like pattern with wreath-like FA lesions and disc leakage, hyperautofluorescent FAF lesions, focal ellipsoid zone disruption on SD-OCT, and multifocal ICGA hypofluorescent spots. A relapse at 6 months with peripapillary inflammatory foci and recurrent cystoid macular edema supported reclassification to a unilateral MFC/PIC-spectrum phenotype. At 2 years, the fellow eye developed mild vitritis, peripapillary hyperautofluorescence, peripapillary/arcade leakage on FA, delayed peripapillary filling on ICGA, and cystoid macular edema, establishing sequential bilateral MFC; no CNV developed and anti-vascular endothelial growth factor (anti-VEGF) therapy was not required. Complications included steroid-induced ocular hypertension and cataract surgery. **Conclusions:** The purpose of this report is to highlight longitudinal imaging “red flags” that supported reclassification from a MEWDS-like phenotype to a sequential bilateral MFC/PIC-spectrum disease.

## 1. Introduction

Multiple evanescent white dot syndrome (MEWDS) is a rare, acute, self-limited outer retinal disorder (estimated incidence ~0.22 per 100,000 population/year) that typically presents unilaterally with characteristic multimodal imaging findings [1,2], whereas idiopathic multifocal choroiditis (MFC) is a chronic, relapsing choriocapillaritis/retinal pigment epithelium (RPE)-centred inflammatory chorioretinopathy that may become bilateral and cause structural complications. We report a MEWDS-like presentation that evolved into sequential bilateral MFC, underscoring diagnostic pitfalls and management implications. This report delineates a MEWDS-like baseline phenotype that was reclassified using SUN criteria during a 4-year multimodal imaging follow-up, culminating in sequential bilateral MFC and illustrating practical diagnostic “red flags” that should prompt closer monitoring and earlier steroid-sparing planning.

## 2. Case Report

A 31-year-old vegetarian woman was referred to ophthalmology from neurology with a provisional diagnosis of presumed retrobulbar optic neuritis after two days of stabbing right-sided headache and sudden profound visual loss in the OD. An external ophthalmologic assessment had documented marked visual acuity reduction with suspected visual field abnormalities. Pattern-reversal visual evoked potentials (VEP) showed reduced amplitudes in the OD with mildly prolonged P100 latency. During the index admission, cranial computed tomography (CT) was unremarkable and lumbar puncture showed clear cerebrospinal fluid (CSF) with 2 cells/µL. Magnetic resonance imaging (MRI) of the brain and cervical/thoracic spine showed no evidence of cerebral demyelination or other acute intracranial pathology; a faint T2-SPAIR hyperintensity at Th1/Th2 was noted, such that myelopathy could not be definitively excluded. High-dose intravenous (IV) methylprednisolone (1 g/day) had been initiated; she was on day two of a planned five-day course. The medical history included treated hypothyroidism and vitamin D/iron deficiency; two months earlier she had received a second Pfizer/BioNTech SARS-CoV-2 vaccination. No causal inference was made, and she reported no intercurrent SARS-CoV-2 infection or systemic symptoms. She otherwise took only a combined oral contraceptive.

At ophthalmic presentation, best-corrected visual acuity (BCVA) was 20/600 OD (right eye) and 20/20 OS (left eye). Intraocular pressure (IOP) measured 18 mmHg bilaterally. IOP was screened using non-contact tonometry (NIDEK) and verified with Goldmann applanation tonometry by the examining physician when clinically indicated. The anterior segment was quiet. Notably, several typical clinical features of optic neuritis were absent, including a relative afferent pupillary defect, red desaturation, pain on eye movements/retropulsion, and optic disc swelling; automated perimetry showed nonspecific defects but was unreliable in the OD due to >85% fixation losses. Dilated fundus examination was unremarkable in both eyes.

Four days into the steroid course, BCVA improved slightly to 20/400 OD, with the OS unchanged and IOP normal. It was planned that the patient would receive one further day of high-dose corticosteroids, to complete the full five-day regimen.

**Figure 1:**
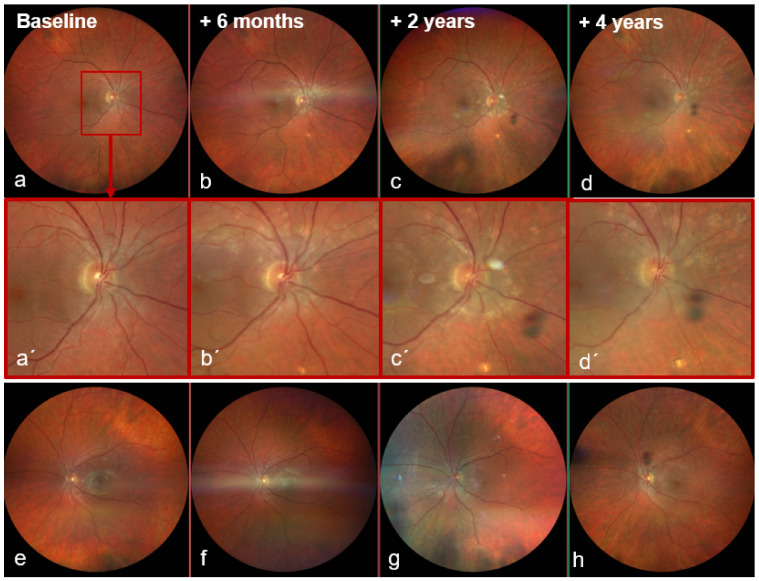
Color fundus photographs (Clarus 500, Zeiss, Oberkochen, Germany). (**a**–**d**) Right eye at baseline (**a**), +6 months (**b**), +2 years (**c**) and +4 years (**d**): baseline shows a discernible foveal reflex; at 6 months, **multiple ill-defined peripapillary chorioretinal inflammatory foci**; +2 years, blurred disc margins with increased peripapillary foci and central cystoid macular edema (CME); +4 years, further increase in peripapillary foci with **chorioretinal scarring and pigment clumping**, particularly inferior to the optic disc. (**a′**–**d′**) Magnified views of the boxed regions. (**e**–**h**) Left eye at the corresponding time-points: baseline (**e**) and +6 months (**f**) with age-appropriate appearance; +2 years (**g**) with **blurred disc margins**, multiple small peripapillary inflammatory spots, CME and two small **dot-shaped hemorrhages** in the superior mid-periphery; +4 years (**h**) showing progression of peripapillary spots, most pronounced superior and nasal to the optic disc.

**Figure 2:**
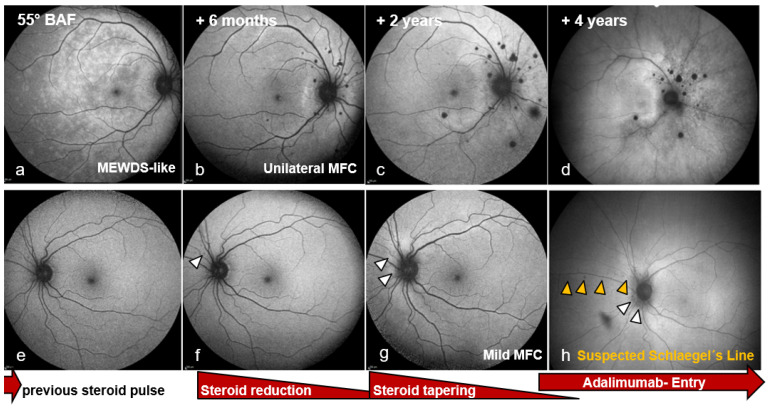
Blue-light fundus autofluorescence (BAF). Spectralis^®^ (Heidelberg Engineering GmbH, Heidelberg, Germany) (**a**–**c**,**e**–**g**); Clarus 500 (ZEISS, Oberkochen, Germany) (**d**,**h**). Right eye (**a**–**d**): baseline—numerous partly confluent **hyperautofluorescent (hyper-AF**) lesions; +6 months—peripapillary round **hypo-AF spots** interspersed with hyper-AF superior/nasal to the disc, a hyper-AF patch in the papillomacular bundle, and confluent **hyper-AF** along the superior arcade; +2 years—clearer peripapillary lesion burden with scar-enlarged hypo-AF areas and progression of confluent hyper-AF along the arcades and nasal to the disc; +4 years—persistent spot burden with apparent blind-spot enlargement, granular peripapillary/arcade hypo-AF, and residual central/papillomacular/superior-arcade hyper-AF. Left eye (**e**–**h**): baseline and +6 months—largely normal; +2 years—peripapillary hyper-AF with expansion of round hypo-AF nasal/superior to the disc and along superior-nasal vessels, plus two hemorrhagic blockage artefacts; +4 years—multiple small peripapillary hypo-AF forming a subtle curved line along the superior-nasal arcade, **suggestive of an early Schlaegel’s line** (**h**). No magnified insets are shown.

**Figure 3:**
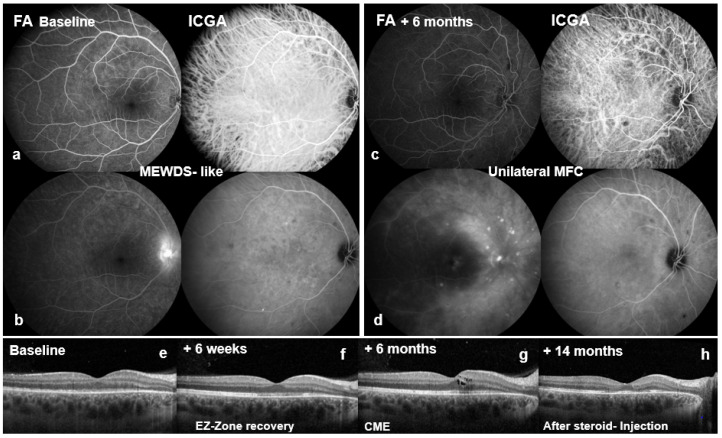
FA/ICGA and SD-OCT of the right eye (Spectralis^®^, Heidelberg Engineering GmbH, Heidelberg, Germany). (**a**,**b**) Baseline (MEWDS-like): FA shows early **wreath-like hyperfluorescence** with late staining and a **hot disc**; ICGA demonstrates multiple **hypofluorescent choriocapillaris spots**. (**c**,**d**) +6 months—reclassification to unilateral MFC: FA reveals numerous early punctate hypofluorescent lesions peripapillary and along the arcades, with late-phase staining and macular leakage; ICGA again shows multiple hypofluorescent dots. (**e**–**h**) SD-OCT: baseline **EZ disruption** (**e**); +6 weeks partial ellipsoid-zone recovery (**f**); +6 months first **CME** (**g**); +14 months dry macula after steroid injection (**h**).

**Figure 4:**
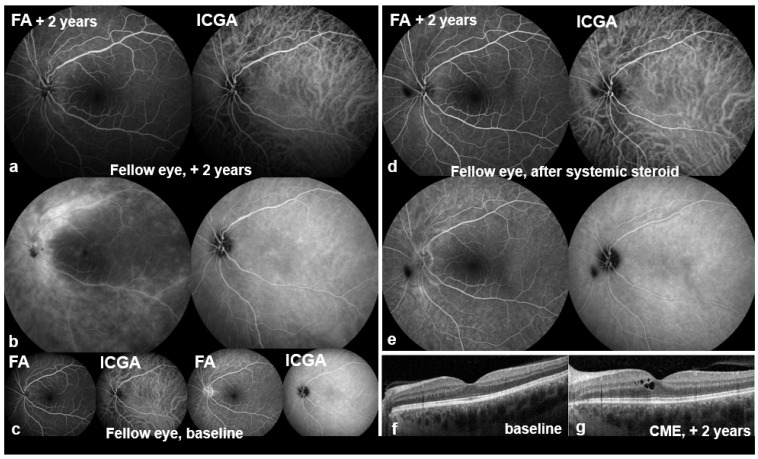
FA/ICGA and SD-OCT of the left eye (Spectralis^®^, Heidelberg Engineering GmbH, Heidelberg, Germany). (**a**,**b**) At 2 years: FA shows marked **peripapillary leakage** spreading along the arcades and centrally; ICGA demonstrates **delayed peripapillary filling** with nasal/superior/temporal **hypofluorescent spots** persisting iso- to hypofluorescent late, with unchanged choroidal vessel calibre. (**c**) Baseline FA/ICGA: FA essentially normal except for a mild hot disc; ICGA shows a few small, nonspecific nasal hypofluorescent dots. (**d**,**e**) Post-steroid: reduced FA leakage with corresponding ICGA improvement. (**f**,**g**) SD-OCT: baseline normal (**f**); first CME at +2 years (**g**). No magnified insets are shown.

Colour fundus photography now showed a sharply demarcated disc and a dry macula without foveal reflex in the OD (Figure 1a), and a healthy disc and macula in the OS (Figure 1e). Multimodal imaging was performed: blue-light fundus autofluorescence (BAF, Spectralis^®^, Heidelberg Engineering GmbH, Heidelberg, Germany) of the OD showed numerous partly confluent hyperautofluorescent lesions (Figure 2a), whereas the OS displayed only two small nasal punctate hypoautofluorescent dots (Figure 2e). Early fluorescein angiography (FA) in the OD demonstrated multiple punctate, wreath-like hyperfluorescent spots—some coalescent—with late leakage and a pronounced hot disc (arm–retina transit time 19 s; Figure 3a,b). FA of the OS was normal apart from mild disc hyperfluorescence. Indocyanine green angiography (ICGA) in the OD disclosed multiple hypofluorescent choriocapillaris spots (Figure 3b,d); the OS showed only a few small nonspecific nasal hypofluorescent areas. Spectral-domain OCT (SD-OCT) revealed focal ellipsoid zone (EZ) disruption in the OD (Figure 3f), with a normal macula in the OS (Figure 4f).

Systemic and neurological investigations were initiated during the index admission. Neurological examination was normal. Infectious testing excluded HSV, VZV, HIV, tick-borne encephalitis, *Borrelia* spp., Mycobacterium tuberculosis, and Treponema pallidum. Aquaporin-4 and myelin oligodendrocyte glycoprotein antibodies were negative. Autoimmune and inflammatory screening showed a low-titer ANA, while ANCAs, lupus anticoagulant, and anticardiolipin antibodies were negative; ACE, soluble interleukin-2 receptor, and lysozyme were within local reference ranges, and serum protein electrophoresis was unremarkable. Owing to the low-titer ANA, the patient was referred to rheumatology at the 6-week follow-up, with no evidence of an underlying systemic rheumatologic disease. Differential diagnoses and the corresponding systemic/neurological investigations are summarized in Figure S3.

At six weeks, BCVA improved to 20/80 OD and remained 20/20 OS. The OD showed three small whitish peripapillary lesions and partial parafoveal EZ recovery on SD-OCT (Figure 3f). The acute unilateral presentation with wreath-like FA lesions and disc leakage, transient outer retinal/EZ disruption on SD-OCT, and absent anterior chamber/vitreous inflammation was consistent with key SUN classification elements for MEWDS [1]. Because foveal granularity and classic grey-white spots could not be objectively documented under the acute circumstances, the baseline phenotype was designated ‘MEWDS-like’ rather than fully classifiable MEWDS and elected close follow-up.

At six months, recurrent visual decline occurred in the OD (BCVA 20/50 OD, 20/25 OS; IOP 13 mmHg both eyes). The OD fundus examination now showed multiple ill-defined peripapillary inflammatory foci, most dense superiorly/nasally, and new cystoid macular edema (CME) (Figure 1b). BAF revealed numerous round hypoautofluorescent peripapillary lesions interspersed with hyperautofluorescent spots, a discrete patch in the papillomacular bundle, and confluent signal along the superior arcade (Figure 2b). FA showed multiple early punctate peripapillary/arcade lesions with late staining and macular leakage; ICGA again disclosed multiple hypofluorescent dots (Figure 3c,d). FA of the OS remained normal, with only mild disc hyperfluorescence; ICGA was likewise unremarkable (Figure 4c). SD-OCT confirmed CME (Figure 3g). Inflammatory CNV was specifically considered and excluded on multimodal imaging (SD-OCT and FA/ICGA); anti-VEGF therapy was not required. Based on the non-monophasic relapse, evolving multifocal peripapillary inflammatory lesions with progressive hypoautofluorescent change, OCT-confirmed CME, and persistent multifocal ICGA hypofluorescent spots, the diagnosis was revised to unilateral MFC within an MFC/PIC spectrum (Table 1; Figure S1).

Therapy was escalated with topical steroidal and non-steroidal drops, parabulbar and intravitreal triamcinolone as needed, and systemic prednisolone (60 mg/day for five days, tapered over twelve weeks to 12.5 mg). Macular edema resolved with a dry macula at 14 months (Figure 3h) but recurred intermittently. Long-acting steroid implants were initially avoided due to phakia. Steroid-induced ocular hypertension developed and was controlled with a triple-agent topical regimen (fixed dorzolamide/timolol combination (Cosopt^®^) plus brimonidine (Alphagan^®^). Immunomodulation was deferred due to pregnancy planning.

Two years after presentation, the OS became symptomatic after two months of intermittent circular photopsias. An examination showed mild vitritis, blurred disc margins, central CME, and two small dot-like superior mid-peripheral hemorrhages (Figure 1g) without macular subretinal exudation; CNV was not evident on SD-OCT or angiography. BCVA remained 20/20 and IOP was normal; OD showed no new acute activity clinically (Figure 1c,c′). OS BAF showed peripapillary hyperautofluorescence with expansion of hypoautofluorescent areas (Figure 2g). FA demonstrated marked peripapillary leakage extending along the arcades (Figure 4a,b). ICGA showed delayed peripapillary filling with nasal/superior/temporal hypofluorescent spots persisting into the late phase; choroidal vessel calibre was unchanged. SD-OCT confirmed CME (Figure 4g). Given that baseline OS FA/ICGA had been essentially normal (Figure 4c), the diagnosis was revised to bilateral MFC, consistent with a chronic relapsing inflammatory trajectory.

Systemic prednisolone induced a rapid anatomical and functional response (Figure 4d,e). Because of the relapsing course, adalimumab 40 mg subcutaneously every two weeks was commenced after twelve weeks, with prednisolone maintained at 12.5 mg daily and then tapered; additional intravitreal triamcinolone was used for recurrences.

By year four, the OD required uncomplicated cataract surgery. Under continued immunomodulation, BAF showed a stable lesion burden in the OD (Figure 2d). In the OS, BAF demonstrated multiple small peripapillary hypoautofluorescent spots forming a subtle curved line along the superior nasal arcade suggestive of an early Schlaegel line (Figure 1h and Figure 2c,f,h). Across follow-up, BCVA in the OD fluctuated between 20/50 and 20/32 in parallel with intraretinal fluid on SD-OCT; the OS remained 20/20. Due to recurrent macular edema in the OD, therapy was ultimately switched to an intravitreal dexamethasone implant. Escalation to dual systemic immunosuppression (adalimumab and methotrexate) was recommended but postponed because of pregnancy planning. No inflammatory CNV developed in either eye during the four-year follow-up. Although OCTA could have provided additional information, repeated dye angiography and structural SD-OCT were considered sufficient to exclude CNV in this case. A summarized treatment timeline is provided in Figure S2.

In summary, an initially MEWDS-like presentation in the OD evolved into unilateral MFC with recurrent CME and was subsequently declared a sequential bilateral disease with fellow-eye involvement at two years.

## 3. Discussion

Phenotypic overlap across white dot syndromes is well recognized, and MEWDS-like presentations may occur within a broader spectrum of inflammatory chorioretinopathies such as MFC/PIC. Sequential or overlapping MEWDS and MFC has been reported for decades, including cases in which MEWDS-like episodes precede MFC-defining lesions or occur in eyes with established MFC, sometimes followed by scarring or CNV [5,6,7,8].

To make our diagnostic labelling transparent, we benchmarked the evolving phenotype against published SUN Working Group classification criteria [1,3,4], acknowledging that these are classification rather than diagnostic criteria. In brief, MEWDS classification requires multifocal grey-white chorioretinal spots with foveal granularity plus characteristic wreath-like FA and/or typical outer retinal OCT lesions, with absent-to-mild anterior chamber/vitreous inflammation, whereas the MFC spectrum reflects true choroidal/chorioretinal inflammatory lesions that may scar, relapse, and develop structural complications (criteria mapping is summarized in Table 1 and visualized in Figure S1). At baseline, foveal granularity and classic grey-white spots were not objectively documented; therefore, we use the term “MEWDS-like”. Published MEWDS-like presentations later reclassified as MFC/PIC are summarized in Table 2; compared with prior reports, which often have shorter follow-up and limited detail on steroid-sparing escalation, our case provides a four-year multimodal imaging and treatment timeline demonstrating a phase shift to chronic-relapsing sequential bilateral MFC, including escalation to adalimumab and an intravitreal dexamethasone implant for recurrent CME.

Consistent with the masquerader framework, Russell et al. emphasized that hyperautofluorescent outer retinal lesions and EZ disruption are not pathognomonic and should prompt caution when the course deviates from the classic self-limited, monophasic pattern [10]. Our case illustrates this: early features were compatible with a MEWDS-like phenotype, yet relapse at six months with evolving peripapillary inflammatory foci and recurrent CME, followed by fellow-eye involvement with vitritis and peripapillary leakage, favoured an MFC/PIC-spectrum trajectory. Similar MEWDS-mimicking patterns in MFC/PIC have been reported, supporting the concept of secondary MEWDS-like reactions within an inflammatory chorioretinopathy rather than a strictly self-limited entity [13].

Sequential bilaterality is also compatible with MFC natural history. In our patient, the OS became involved after two years. Fung et al. reported that approximately 50% present bilaterally at baseline and approximately 25% of initially unilateral cases develop contralateral involvement within five years [12]. This context supports interpreting delayed fellow-eye involvement as recognized behaviour within the MFC spectrum.

Importantly, ICGA hypofluorescence is not specific to MFC/PIC and should not be used as a standalone discriminator from MEWDS. In MEWDS, both ICGA hypofluorescent spots and OCTA findings at the choriocapillaris level have been reported with heterogeneous results [17,18], and swept-source OCTA studies have also described transient, reversible choriocapillaris flow deficits during acute MEWDS that resolve with clinical recovery [19]. Cohort data comparing MEWDS with versus without overlapping MFC (including OCTA) further support this heterogeneity and report suspected choriocapillaris flow deficits in overlap cases, with later development of MFC lesions in the affected or fellow eye [8]. OCTA was not performed in this case, and quantitative OCTA/ICGA metrics were therefore not available. We recommend incorporating OCTA in future MEWDS-like/MFC-overlap presentations to assess choriocapillaris flow non-invasively and complement dye angiography. Accordingly, our conclusions do not require that MEWDS universally involves the choriocapillaris; in our patient, the diagnostic weight derived from the longitudinal multimodal pattern—relapse, progressive peripapillary inflammatory activity, recurrent CME, later vitritis/peripapillary leakage in the fellow eye, and persistent ICGA abnormalities—supported an evolving inflammatory chorioretinopathy. Practically, ICGA can be framed as a risk-stratifying modality in MEWDS-like presentations: extensive or persistent hypofluorescent spots, particularly when paired with recurrence, evolving hypoautofluorescence on FAF, vitreous activity, peripapillary inflammatory progression, or CME, should prompt heightened suspicion for an MFC/PIC-spectrum diagnosis and closer follow-up; early hypoautofluorescence may also indicate a more chronic inflammatory chorioretinopathy in the masquerader literature [10].

MEWDS-like presentations may be referred as neuro-ophthalmic disease when profound acute vision loss and optic disc leakage dominate, particularly when accompanied by reported visual field abnormalities and supportive (but non-specific) VEP changes. In our case, a high-dose IV corticosteroid pulse was administered for presumed retrobulbar neuritis, reflecting a recognized diagnostic pitfall; similar misclassifications have been reported [15,16]. While pulse steroids could theoretically modulate lesion visibility, causality remains speculative.

Finally, the therapeutic course provides pragmatic implications. Early BCVA improvement coincided with partial EZ restoration; later fluctuations mirrored OCT-confirmed CME. Recurrent CME drove morbidity and necessitated repeated corticosteroid exposure, culminating in predictable steroid-related sequelae (ocular hypertension and cataract surgery). Because inflammatory CNV is a major cause of exudation and vision loss in the MFC/PIC spectrum [14], CNV must be actively assessed whenever macular fluid recurs; in our patient, repeated episodes represented OCT-confirmed CME without multimodal evidence of CNV, and anti-VEGF therapy was therefore not required. These real-world trade-offs underscore the need to consider earlier steroid-sparing strategies in relapsing MFC/PIC rather than repeated cycles of local/systemic corticosteroids. The escalation to adalimumab is supported by the VISUAL I/II trials, which demonstrated reduced relapse risk and improved disease control in noninfectious intermediate uveitis, posterior uveitis, and panuveitis [20,21]. Recurrent CME was ultimately managed with an intravitreal dexamethasone implant, consistent with HURON trial evidence in non-infectious posterior uveitis, albeit with predictable adverse effects [22]. Intravitreal triamcinolone remains effective for uveitic CME but is limited by a shorter duration and frequent IOP elevation [23].

## 4. Conclusions

A MEWDS-like onset may be the first sign of an evolving MFC spectrum and can conceal a chronic-relapsing course with delayed fellow-eye involvement. Serial multimodal imaging is crucial to detect evolution early, support timely reclassification, and guide steroid-sparing management, with targeted local therapy for CME and vigilant monitoring for steroid-related ocular hypertension and cataract. When such cases are referred as presumed retrobulbar neuritis, structured interdisciplinary evaluation helps prevent misdiagnosis; MEWDS-like presentations that relapse, develop CME, or show progressive FAF changes should be managed as potential MFC masqueraders and followed accordingly. Further studies are needed to refine mechanisms and imaging-guided management.

## Figures and Tables

**Table 1:** Criteria-based justification of “MEWDS-like” labelling at onset and subsequent reclassification to an MFC/PIC-spectrum phenotype (SUN classification framework).

Domain/Criterion	MEWDS (SUN) [1]	PIC (SUN) [3]	MFCPU (SUN) [4]	Our Case at Presentation (OD)	Our Case Over Time (OD, +6 Months–+4 Years) and Fellow Eye (OS, +2 Years)
Core phenotype	Acute, typically self-limited outer retinopathy	Punctate inner choroidopathy (punctate choroidal lesions)	MFC with panuveitis	MEWDS-like multimodal pattern at onset	Relapsing inflammatory chorioretinal disease consistent with an MFC/PIC-spectrum phenotype
Foveal granularity	Core classification element	Not required	Not required	Not objectively documented at baseline (hence “MEWDS-like”)	Not defining; later course driven by chorioretinal inflammatory signs
FA	Wreath-like lesions; +/− disc leakage	Active lesions may stain/leak	Inflammatory lesions and/or peripapillary/arcade leakage may be present	Wreath-like punctate hyperfluorescent lesions and hot disc	Peripapillary/arcade leakage and macular leakage with CME; OS: marked peripapillary leakage along arcades
FAF/BAF	Multiple hyperautofluorescent dots/spots	Mixed FAF changes; hypoautofluorescent lesions/scars may develop	Progressive hypoautofluorescent lesions/scars may develop	Numerous partly confluent hyperautofluorescent lesions	Evolution to peripapillary hypoautofluorescent lesions; OS: peripapillary hyper-AF with expanding hypo-AF
SD-OCT	Transient outer retinal/EZ disruption with recovery	Focal outer retinal/RPE lesions; CNV risk	Chorioretinal lesions/scars; complications may occur	Focal EZ disruption with partial early recovery	Recurrent CME on OCT; fluctuating intraretinal fluid; OS: OCT-confirmed CME at onset
AC/vitreous	Absent to mild inflammation	Absent or minimal inflammation	More than minimal inflammation and/or scars (classification dependent)	Quiet anterior segment; no vitritis at baseline	OS: mild vitritis at onset; later scar/pigmentary change OD
Course	Usually monophasic, self-limited	Variable; can recur	Typically, chronic relapsing	Early partial recovery at 6 weeks	Relapses at 6 months with recurrent activity; sequential fellow-eye involvement at 2 years
Structural complications	Complications uncommon; CME not typical	CNV common; other sequelae may occur	CME/CNV and other complications may occur	None at baseline	Recurrent CME; no CNV; steroid-induced ocular hypertension and cataract; escalation to immunomodulation and intravitreal steroid implant
ICGA	Hypofluorescent spots may occur; not diagnostic alone	Hypofluorescent choriocapillaris spots may be present	Hypofluorescent choriocapillaris spots may be present	Multiple hypofluorescent choriocapillaris spots	Persistent/recurrent hypofluorescent spots; OS: delayed peripapillary filling with hypofluorescent spots
Bilaterality	Usually, unilateral	May be bilateral	Often bilateral or becomes bilateral over time	Unilateral at baseline	Sequential bilaterality with fellow-eye involvement at 2 years

**Table 2:** Reported cases and series with MEWDS-like presentation later reclassified as MFC/PIC.

Author (Year)	No. of Cases	Initial Diagnosis	Final Diagnosis/Course	Key Imaging Markers	Time to Bilaterality	Therapy	Outcome
Jampol et al. (1984) [9]	≈10 (classic series)	Classical MEWDS (foveal granularity, wreath-like FA, disc leakage)	Self-limited MEWDS; no transition to MFC	FA: wreath-like; BAF: multiple hyper-AF spots	Rare	Observation	Spontaneous recovery of function
Russell et al. (2020) [10]	7	MEWDS-like features (BAF hyper-AF, EZ loss)	Various conditions incl. MFC (MEWDS ‘masqueraders’)	BAF pattern not pathognomonic; OCT: EZ disruption	Variable	Heterogeneous, per underlying disease	Depends on underlying diagnosis
Munk et al. (2015) [11]	5	MEWDS-like with acute central photoreceptor dysfunction	MFC/PIC with discrete lesions	OCT: EZ loss disproportionate to visible lesions	1–3 years in 2 cases (reported)	Systemic steroids; immunosuppression	Stabilization; CNV in subset
Fung et al. (2014) [12]	41	Heterogeneous; some MEWDS-like	MFC (often bilateral over time)	ICGA: multiple hypofluorescent choriocapillaris spots	≈25% within 60 months	Corticosteroids; immunosuppression	Bilateral involvement and CNV in a subset
Chen et al. (2024) [13]	6	MEWDS-like (multiple BAF spots, EZ disruption)	Juxtafoveal MFC/PIC	Rapid disappearance of BAF dots; EZ re-normalization	2/6 bilateral (months–years)	Corticosteroids; immunosuppression	Generally good vision; CNV risk persists
Borrego-Sanz et al. (2019) [14]	72 (3 centres)	White dot syndromes (incl. MEWDS, MFC)	Chronic MFC/PIC with relapses in subset	CME and CNV drive prognosis	≈30%	Steroids; IS; anti-VEGF as needed	Vision strongly depends on complications
Pellegrini (2016) [15]; Han (2025) [16]	2 (case reports)	Atypical optic neuritis (misdiagnosis)	MEWDS; later course consistent with MFC/PIC in some reports	FA: hot disc; OCT: EZ loss; ICGA: focal spots (when performed)	Not reported	High-dose steroids initially; later IS per final diagnosis	Improvement after reclassification
Present case (2026)	Single case report	presumed retrobulbar optic neuritis (misdiagnosis), then MEWDS-like OD	Sequential bilateral MFC	FA: wreath-like; FAF hyperautofluorescent; EZ focal disruption; ICGA multifocal spots; OCT: longitudinal phase shift, CME, no CNV	2 years	IV high-dose steroids initially; then topical, periocular, intravitreal, and systemic corticosteroids, later adalimumab (steroid-sparing)/Ozurdex^®^	Chronic relapsing, OD fluctuating BCVA with recurrent CME; OS preserved; steroid IOP rise, cataract surgery; recommendation dual systemic IS

## Data Availability

The datasets generated and/or analyzed during the current study are not publicly available due to patient confidentiality and German data protection regulations. However, the data may be made available by the corresponding author upon request and with permission from the responsible institutional review board of Eye Clinic Sulzbach.

## Supplementary Materials

The following supporting information can be downloaded at: https://www.mdpi.com/article/10.3390/biomedicines14030649/s1, Figure S1: SUN criteria timeline matrix; Figure S2: Treatment timeline; Figure S3: Differential diagnosis and systemic/neurological work-up.

## Abbreviations

ACE	Angiotensin-converting enzyme
AC	Anterior chamber
ANA	Antinuclear antibody
ANCA	Anti-neutrophil cytoplasmic antibody
BAF	Blue-light autofluorescence
BCVA	Best corrected visual acuity
CME	Cystoid macular edema
CNV	Choroidal neovascularization
CSF	Cerebrospinal fluid
CT	Computed tomography
EZ	Ellipsoid zone
FA	Fluorescein angiography
FAF	Fundus autofluorescence
HIV	Human immunodeficiency virus
HSV	Herpes simplex virus
ICGA	Indocyanine green angiography
IOP	Intraocular pressure
IS	Immunosuppression
IV	Intravenous
MFCPU	Multifocal choroiditis with panuveitis
MEWDS	Multiple evanescent white dot syndrome
MFC	Multifocal choroiditis
MOG	Myelin oligodendrocyte glycoprotein
MOGAD	Myelin oligodendrocyte glycoprotein antibody-associated disease
MRI	Magnetic resonance imaging
MS	Multiple sclerosis
NMOSD	Neuromyelitis optica spectrum disorder
OCT	Optical coherence tomography
OD	Right eye
OS	Left eye
PIC	Punctate inner choroidopathy
PRVEP	Pattern reversal visual evoked potentials
RPE	Retinal pigment epithelium
SD-OCT	Spectral-domain optical coherence tomography
SUN	Standardization of Uveitis Nomenclature
TNF	Tumour necrosis factor
VEP	Visual evoked potentials
VZV	Varicella-zoster virus

## References

[1] (2021). The Standardization of Uveitis Nomenclature (SUN) Working Group. Classification Criteria For Multiple Evanescent White Dot Syndrome. Am. J. Ophthalmol..

[2] Abu-Yaghi N.E., Hartono S.P., Hodge D.O., Pulido J.S., Bakri S.J. (2011). White dot syndromes: A 20-year study of incidence, clinical features, and outcomes. Ocul. Immunol. Inflamm..

[3] (2021). The Standardization of Uveitis Nomenclature (SUN) Working Group. Classification Criteria for Punctate Inner Choroiditis. Am. J. Ophthalmol..

[4] (2021). The Standardization of Uveitis Nomenclature (SUN) Working Group. Classification Criteria for Multifocal Choroiditis with Panuveitis. Am. J. Ophthalmol..

[5] Callanan D., Gass J.D. (1992). Multifocal choroiditis and choroidal neovascularization associated with the multiple evanescent white dot and acute idiopathic blind spot enlargement syndrome. Ophthalmology.

[6] Kuznetcova T., Jeannin B., Herbort C.P. (2012). A case of overlapping choriocapillaritis syndromes: Multimodal imaging appraisal. J. Ophthalmic Vis. Res..

[7] Bryan R.G., Freund K.B., Yannuzzi L.A., Spaide R.F., Huang S.J., Costa D.L. (2002). Multiple evanescent white dot syndrome in patients with multifocal choroiditis. Retina.

[8] Kang H.G., Kim T.Y., Kim M., Byeon S.H., Kim S.S., Koh H.J., Lee S.C., Lee C.S. (2022). Expanding the Clinical Spectrum of Multiple Evanescent White Dot Syndrome with Overlapping Multifocal Choroiditis. Ocul. Immunol. Inflamm..

[9] Jampol L.M., Sieving P.A., Pugh D., Fishman G.A., Gilbert H. (1984). Multiple evanescent white dot syndrome. I. Clinical findings. Arch. Ophthalmol..

[10] Russell J.F., Pichi F., Scott N.L., Hartley M.J., Bell D., Agarwal A., Leong B., Holland G.N., Freund K.B., Sarraf D. (2020). Masqueraders of multiple evanescent white dot syndrome (MEWDS). Int. Ophthalmol..

[11] Munk M.R., Jung J.J., Biggee K., Tucker W.R., Sen H.N., Schmidt-Erfurth U., Fawzi A.A., Jampol L.M. (2015). Idiopathic multifocal choroiditis/punctate inner choroidopathy with acute photoreceptor loss or dysfunction out of proportion to clinically visible lesions. Retina.

[12] Fung A.T., Pal S., Yannuzzi N.A., Christos P., Cooney M., Slakter J.S., Klancnik J.M., Freund K.B., Cunningham E.T., Yannuzzi L.A. (2014). Multifocal choroiditis without panuveitis: Clinical characteristics and progression. Retina.

[13] Chen C., Cheng Y., Zhang Z., Zhang Y., Hou S., Wang G., Peng X. (2024). The multimodal imaging features and outcomes of multifocal choroiditis/punctate inner choroidopathy lesion with multiple evanescent white dot syndrome-like features: A retrospective study. BMC Ophthalmol..

[14] Borrego-Sanz L., Gomez-Gomez A., Gurrea-Almela M., Esteban-Ortega M., Pato E., Diaz-Valle D., Diaz-Valle T., Munoz-Fernandez S., Rodriguez-Rodriguez L., Madrid Uveitis Study G. (2019). Visual acuity loss and development of ocular complications in white dot syndromes: A longitudinal analysis of 3 centers. Graefes Arch. Clin. Exp. Ophthalmol..

[15] Pellegrini F., Interlandi E. (2016). A case of multiple evanescent white dot syndrome misdiagnosed as optic neuritis: Differential diagnosis for the neurologist. J. Neurosci. Rural. Pract..

[16] Han K.E., Lee S.M., Kim S.J., Choi H., Choi J.H. (2025). Multiple evanescent white dot syndrome masquerading as atypical optic neuritis: A case report. J. Med. Case Rep..

[17] Yannuzzi N.A., Swaminathan S.S., Zheng F., Miller A., Gregori G., Davis J.L., Rosenfeld P.J. (2017). Swept-Source OCT Angiography Shows Sparing of the Choriocapillaris in Multiple Evanescent White Dot Syndrome. Ophthalmic Surg. Lasers Imaging Retin..

[18] Pereira F., Lima L.H., de Azevedo A.G.B., Zett C., Farah M.E., Belfort R. (2018). Swept-source OCT in patients with multiple evanescent white dot syndrome. J. Ophthalmic Inflamm. Infect..

[19] Khochtali S., Dridi T., Abroug N., Ksiaa I., Lupidi M., Khairallah M. (2020). Swept-Source Optical Coherence Tomography Angiography Shows Choriocapillaris Flow Reduction in Multiple Evanescent White Dot Syndrome. J. Curr. Ophthalmol..

[20] Jaffe G.J., Dick A.D., Brezin A.P., Nguyen Q.D., Thorne J.E., Kestelyn P., Barisani-Asenbauer T., Franco P., Heiligenhaus A., Scales D. (2016). Adalimumab in Patients with Active Noninfectious Uveitis. N. Engl. J. Med..

[21] Nguyen Q.D., Merrill P.T., Jaffe G.J., Dick A.D., Kurup S.K., Sheppard J., Schlaen A., Pavesio C., Cimino L., Van Calster J. (2016). Adalimumab for prevention of uveitic flare in patients with inactive non-infectious uveitis controlled by corticosteroids (VISUAL II): A multicentre, double-masked, randomised, placebo-controlled phase 3 trial. Lancet.

[22] Lowder C., Belfort R., Lightman S., Foster C.S., Robinson M.R., Schiffman R.M., Li X.Y., Cui H., Whitcup S.M., Ozurdex H.S.G. (2011). Dexamethasone intravitreal implant for noninfectious intermediate or posterior uveitis. Arch. Ophthalmol..

[23] Antcliff R.J., Spalton D.J., Stanford M.R., Graham E.M., Ffytche T.J., Marshall J. (2001). Intravitreal triamcinolone for uveitic cystoid macular edema: An optical coherence tomography study. Ophthalmology.

