# Supplementary material for: MEWDS-like Presentation Unmasking Sequential Bilateral Multifocal Choroiditis: Insights from Longitudinal Multimodal Imaging
Source: Biomedicines. 2026 Mar 13;14(3):649. doi: 10.3390/biomedicines14030649 (PMC13024056; doi:10.3390/biomedicines14030649)
Supplement: Supplementary file 1 [file biomedicines-14-00649-s001.zip › biomedicines-4171313-supplementary.pdf]

SUN criteria / features	Baseline (Day 4)	6 weeks	6 months	2 years (OS onset)	4 years
**MEWDS (SUN)**					
Gray-white spots	ND	~	✗	✗	✗
Foveal granularity	ND	ND	ND	ND	ND
Wreath-like FA	✓	ND	✗	✗	✗
Outer retinal OCT lesion	✓	✓	~	~	~
Absent/mild inflammation	✓	✓	✓	~	~
**PIC (SUN)**					
Punctate lesions <250 µm	ND	ND	✓	✓	✓
Minimal/no AC/vitreous inflammation	✓	✓	✓	~	~
Posterior pole ± mid-periphery	ND	ND	✓	✓	✓
**MFCPU (SUN)**					
MFC lesions >125 µm	✗	~	✓	✓	✓
Lesions outside posterior pole	✗	✗	✓	✓	✓
Punched-out scars or >minimal vitritis	✗	✗	✓	✓	✓
**Course / complications**					
Non-monophasic relapse	✗	✗	✓	✓	✓
CME	✗	✗	✓	✓	✓
Fellow-eye involvement	✗	✗	✗	✓	✓
CNV (assessed)	✗	✗	✗	✗	✗
**Working label**	MEWDS-like	MEWDS-like	Unilateral MFC	Bilateral MFC	Bilateral MFC

Figure S1. (Supplement 1). SUN criteria timeline matrix. Presence (✓), absence (✗), lack of documentation (ND), or atypical/partial fulfillment (~) of key SUN Working Group classification elements over time. The bottom row indicates the evolving working clinical label (MEWDS-like → unilateral MFC → bilateral MFC).

Timepoint	Eye	Treatment (route; dose)	Key outcome / notes
Day 0-5	OD	IV methylprednisolone 1 g/day x 5 days	Started for presumed retrobulbar optic neuritis; early partial visual improvement
Month 6 (relapse) to month 24	OD	Topical steroid + NSAID; parabulbar triamcinolone (Volon A) and intravitreal triamcinolone as needed; oral prednisolone 60 mg/day x 5 days then taper to 12.5 mg/day maintenance	CME improved (dry macula at month 14) but recurred intermittently. Steroid-induced ocular hypertension controlled with dorzolamide/timolol + brimonidine; long-acting implants initially avoided (phakic); immunomodulation deferred due to pregnancy planning
Year 2 (OS onset) + 12 weeks	OS / Both	Systemic prednisolone for OS onset; adalimumab 40 mg SC every 2 weeks started after 12 weeks; prednisolone 12.5 mg/day then taper; intravitreal triamcinolone for recurrences	Rapid response to systemic steroids; relapsing course prompted steroid-sparing escalation
Year 4	OD (±Both)	Cataract surgery (OD); intravitreal dexamethasone implant (Ozurdex) for recurrent CME; dual systemic therapy (adalimumab + methotrexate) recommended but postponed	OD vision fluctuated with intraretinal fluid; OS remained 20/20

Figure S2. Treatment timeline.

Domain / differential diagnosis	Investigations performed	Result
Demyelinating disease (MS/NMOSD/MOGAD)	MRI brain and cervical/thoracic spine; CSF analysis (cell count, oligoclonal bands); aquaporin-4 antibodies; myelin oligodendrocyte glycoprotein antibodies	No cerebral demyelination or acute intracranial pathology; faint T2-SPAIR hyperintensity at Th1/Th2 (clinical myelopathy not confirmed); CSF clear with 2 cells/μL and no oligoclonal bands; aquaporin-4 and MOG antibodies negative
Infectious etiologies (e.g., herpetic disease, HIV, TB, syphilis)	Serum/CSF testing for HSV, VZV, HIV, tick-borne encephalitis, Borrelia spp., Mycobacterium tuberculosis, Treponema pallidum	Negative
Systemic granulomatous/inflammatory disease (e.g., sarcoidosis)	ACE, soluble interleukin-2 receptor, lysozyme	Within local reference ranges
Systemic autoimmune/vasculitic disease	ANA; ANCAs; lupus anticoagulant; anticardiolipin antibodies	Low-titer ANA; ANCAs, lupus anticoagulant and anticardiolipin antibodies negative
Hematologic	Serum protein electrophoresis	Unremarkable
Rheumatologic assessment	Clinical rheumatology evaluation following low-titer ANA	No evidence of underlying systemic rheumatologic disease
Acute intracranial pathology (index admission)	Cranial CT	Unremarkable

Figure S3. Differential diagnosis and systemic/neurological work-up.
